# Relational Communication Competence in Nursing Leadership and Management: A Concept Analysis

**DOI:** 10.1155/jonm/1364784

**Published:** 2026-07-30

**Authors:** Joseph Almazan, Darya Zvonareva, Eddieson Pasay-an, Cris Adolfo, Cyrelle Agunod, Srinivasa Rao Bolla

**Affiliations:** ^1^ Department of Medicine, School of Medicine, Nazarbayev University, Kerey and Zhanibek Khans St 5/1, Astana 010000, Kazakhstan, nu.edu.kz; ^2^ Teaching and Learning Support Office, NU Library, Nazarbayev University, 53 Kabanbay Batyr Avenue, Astana, Kazakhstan, nu.edu.kz; ^3^ College of Nursing, King Khalid University, Abha, Saudi Arabia, kku.edu.sa; ^4^ Nursing Department, St. André Health Care, Biddeford, Maine, USA; ^5^ Nursing Department, North Private College of Nursing, Arar, Saudi Arabia; ^6^ Department of Biomedical Sciences, School of Medicine, Nazarbayev University, Kerey and Zhanibek Khans St 5/1, Astana, Kazakhstan, 010000, nu.edu.kz

**Keywords:** concept analysis, nursing leadership, nursing management, older adults, person-centered communication, relational communication competence

## Abstract

**Aim:**

To clarify the concept of relational communication competence (RCC) within nursing leadership and management through concept analysis.

**Background:**

No unified conceptual definition currently bridges leadership, communication competence, and person‐centered care in nursing, a gap that constrains both competency frameworks and workforce development. While the issue is illustrated through the context of older‐adult care, the analysis extends more broadly to nursing leadership and management across diverse clinical and organizational settings.

**Methods:**

We conducted a concept analysis guided by Walker and Avant’s framework. A structured search across PubMed, Scopus, Web of Science, and APA PsycNet yielded 19 eligible studies. Two reviewers independently extracted conceptual definitions, communication behaviors, and theoretical constructs from each study into a structured concept matrix, then compared entries iteratively until recurring patterns consolidated into defining attributes, antecedents, consequences, and empirical referents, with no new attributes emerging from the final studies reviewed.

**Results:**

The analysis identified three defining attributes: relational sensitivity, effective information exchange, and collaborative engagement. All three attributes must co‐occur: No single attribute alone constitutes RCC, and this combination has not been consistently specified in nursing leadership scholarship. Antecedents included communication knowledge, cultural awareness, structured dialog opportunities, and supportive organizational climates. Consequences included stronger therapeutic relationships, greater patient engagement, improved team collaboration, and reduced clinical uncertainty. Empirical referents included empathic listening, transparent clinical explanation, verified shared understanding, and facilitated dialog.

**Conclusion:**

RCC is defined as the capacity for healthcare communication that is responsive to contextual and emotional cues, informationally clear, and participatory across patients, families, and professionals. The analysis offers an integrated framework for bringing relational leadership, communication competence, and person‐centered care into closer conceptual alignment in nursing practice.

**Implications for Nursing Management:**

Nurse managers require programs that develop all three attributes together, embedding RCC as a unified leadership capability rather than treating awareness, information exchange, and participation as separate concerns.

## 1. Background

Communication is central to nursing practice and leadership, shaping interactions among healthcare professionals, patients, and families across diverse healthcare systems and cultural contexts [1, 2]. For nurses in both leadership and management roles, communication involves more than transmitting clinical information; it includes the relational processes through which trust, respect, and collaboration develop. Nurse leaders shape the relational culture of care environments, while nurse managers hold direct responsibility for workforce supervision, team coordination, and operational outcomes, yet both roles depend substantially on relational communication to function effectively.

Healthcare communication operates across informational and relational dimensions. Informational communication concerns the exchange of clinical knowledge and care instructions, while relational communication reflects how empathy, attentiveness, and respect are expressed during clinical encounters ([3], p. 4). Poor communication within healthcare teams is implicated in the majority of sentinel events reported across acute care settings [4]. Research shows that relational messages embedded in clinical interactions influence patient engagement, satisfaction, and participation in care decisions [5, 6], while responsiveness to patients’ relational and emotional needs is central to effective therapeutic relationships [7, 8]. These findings indicate that communication competence in healthcare requires both technical skill and the capacity to establish meaningful interpersonal relationships that support patient safety and care quality.

This relational dimension is especially important in the care of older adults, a clinically relevant motivating example rather than a constraint on the analytical scope. The inclusion criteria specified healthcare contexts broadly; the findings apply across clinical and organizational nursing settings. According to the United Nations Department of Economic and Social Affairs ([9], p. 1), the number of persons aged 65 or over is projected to more than double, reaching over 1.5 billion by 2050, with the majority presenting with multiple chronic conditions, cognitive decline, and care trajectories spanning multiple settings and transitions [10–12]. Older adults often depend on healthcare professionals to interpret complex medical information while preserving autonomy and dignity, making relational communication central to person‐centered care [13]. Research suggests that relational engagement, including honest dialog and culturally responsive interaction, supports trust and meaningful involvement in care decisions among this population [14, 15]. This context positions nursing leadership communication as an organizational responsibility closely linked to care quality for a population with complex and sustained care needs.

Within nursing, communication competence is central to effective leadership and management practice. Nurse leaders and managers shape communication patterns and the relational environment within their teams, influencing care coordination, clinical decision‐making, and interpersonal dynamics across complex organizational settings [1, 16]. Relational communication behaviors contribute to stronger team cohesion, more constructive conflict management, and better‐coordinated patient care [2, 17]. Research on interdisciplinary teams similarly shows that communication patterns shape clinical decision‐making and care coordination outcomes [18]. These findings establish communication competence as a relational capability fundamental to nursing leadership and management.

Despite this recognized importance, the relational dimension of communication competence in nursing leadership presents two distinct problems: It remains poorly defined as a concept, and the literature addressing it is theoretically fragmented across disciplines. To date, nursing practice has received limited attention regarding the connection between these dimensions. Interpersonal communication research defines communication competence as the ability to communicate effectively and appropriately in specific situations ([19], p. 7), yet this definition has not been consistently applied in nursing contexts. Relational leadership scholarship understands leadership as emerging through interaction and mutual influence [20], while nursing scholarship has separately advanced person‐centered care frameworks that place relational engagement at the center of effective practice [13, 15]. Although these three bodies of work share a common relational orientation, each has developed largely in isolation. Communication Competence Theory ([19], p. 7) does not integrate relational leadership or person‐centered care values; Relational Leadership Theory [20] does not specify the communicative behaviors through which relational leadership is enacted; and Person‐Centered Care Frameworks [13] do not operationalize the communicative processes through which person‐centered relationships are enacted in nursing leadership. A central contradiction within this literature is that some studies treat communication competence as an individual trait trainable in isolation, while others position it as a relational process emerging through interaction, a distinction with direct consequences for how relational communication competence (RCC) is developed, assessed, and supported in nursing leadership.

As a result, the connections between communication competence, leadership, and person‐centered care in nursing remain poorly integrated [21], limiting the development of competency frameworks and workforce development initiatives that require a clear account of relational communication in nursing practice. Clarifying the conceptual structure of RCC in nursing leadership, therefore, addresses a gap with direct consequences for practice: Without a coherent and integrated account of what RCC means in nursing leadership and management, efforts to develop, assess, and improve communication within healthcare teams lack a shared theoretical foundation.

RCC is used in this study as a provisional working concept, understood as communication that is empathic, contextually responsive, and relationship‐sustaining in professional care settings; this formulation is subject to refinement through the analysis. Given the absence of a consensus definition and its inconsistent operationalization across nursing, leadership, and communication literature, this study seeks to clarify the conceptual structure and defining characteristics of RCC through concept analysis.

## 2. Theoretical Foundation

This concept analysis is guided by three complementary frameworks, each addressing a distinct dimension of RCC while sharing the premise that professional relationships, rather than individual competencies or task performance, are central to effective healthcare practice.

Communication Competence Theory [19, 21] defines competence as behavior that is both contextually appropriate and goal‐effective, distinguishing clinically accurate communication from relationally competent interaction.

Person‐Centered Practice Framework ([13], pp. 3–25; [22], pp. 13–27) emphasizes empathic engagement, respect for individual values, and collaborative decision‐making as the foundation of nursing practice, directly linking relational communication to care outcomes.

Relational Leadership Theory [20] interprets leadership as a process built through trust, mutual respect, and collaboration, showing how nurse leaders shape the relational conditions that enable teams to coordinate care.

Together, these frameworks demonstrate that RCC functions across the relational climate leaders foster, the communication behaviors enacted in clinical interactions, and the person‐centered values guiding those interactions. They provide the theoretical basis for identifying the defining attributes, antecedents, and consequences examined in this analysis.

## 3. Aim

To clarify the concept of RCC within nursing leadership and management through concept analysis.

## 4. Objectives

1. To examine how RCC and related constructs are conceptualized and defined across nursing, leadership, and healthcare communication literature.
2. To identify the defining attributes of RCC within nursing leadership and healthcare communication contexts.
3. To determine the antecedents, consequences, and empirical referents associated with RCC in healthcare interactions.
4. To construct a conceptual definition of RCC that integrates relational leadership, communication competence, and person‐centered care perspectives, applicable to nursing leadership and management practice.

## 5. Methods

The three theoretical frameworks grounding this analysis are described in the Background. The analysis itself was conducted using Walker and Avant’s [23] eight‐step concept analysis framework, an established method for clarifying concepts that are ambiguously defined or inconsistently operationalized across the literature [23, 24].

### 5.1. Design

This study employed concept analysis guided by Walker and Avant’s [23] methodological framework. Concept analysis is an established method in nursing scholarship for clarifying concepts that are ambiguously defined or inconsistently operationalized across the literature [23, 24]. This method was selected because the aim of this study was not to synthesize empirical evidence but to clarify the conceptual structure of RCC, a concept that lacks a consensus definition and has been operationalized differently across nursing, leadership, and communication literature. Walker and Avant’s [23] approach was selected over Rodgers’ [24] evolutionary concept analysis because the objective was definitional clarification of a poorly bounded construct rather than tracing its historical conceptual evolution. Concept analysis is designed for theoretical and conceptual clarification; the findings do not constitute empirical hypotheses and should not be evaluated against criteria for statistical generalization. Walker and Avant [23] identify precisely these conditions as the primary indication for concept analysis. The framework provides a structured eight‐step process for examining how a concept is used in literature, identifying its defining attributes, antecedents, consequences, and empirical referents. The primary methodological limitation of Walker and Avant’s framework, that the researcher’s professional orientation may influence attribute identification, was mitigated through independent coding by two reviewers prior to consensus discussions.

### 5.2. Data Sources and Search Strategy

We conducted a structured literature search to capture uses, meanings, and applications of RCC and related constructs within healthcare communication and nursing scholarship. To ensure transparency, elements of the PRISMA 2020 framework were adapted to guide identification and selection [25]. Because this study is a concept analysis rather than a formal systematic review, PRISMA components were applied selectively and in alignment with concept‐analysis requirements. Searches were performed across four electronic databases (PubMed, Scopus, Web of Science, and APA PsycNet), yielding 4317 records. Fully parenthesized database‐specific strategies, applied filters, and records retrieved are provided in Supporting Table S1.

The core search combined relational communication terms (“relational communication” OR “communication competence”) with nursing leadership and healthcare‐context terms (“nursing leadership” OR “nurse manager” OR “patient‐centered care” OR “older adults”). Database‐specific field tags and filters were then applied according to each platform (Supporting Table S1).

The search covered publications from January 2000 to December 2024. The year 2000 was selected as the primary cutoff because Relational Leadership Theory [20] and contemporary Person‐Centered Care Frameworks [13] gained broad adoption during this period; pre‐2000 foundational theoretical works were included by two‐reviewer consensus where directly relevant. Reference lists of included articles were manually screened to identify additional conceptually relevant sources.

**FIGURE 1:**
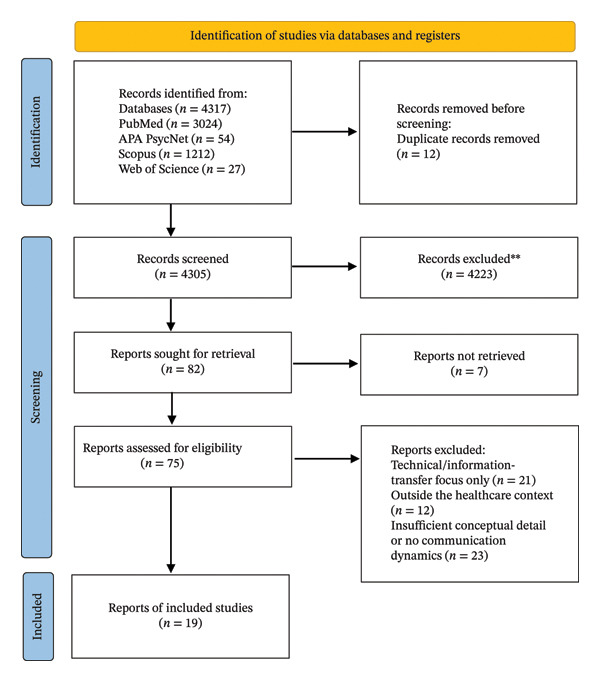
PRISMA‐adapted flow diagram of the study selection process. Note. Adapted from the PRISMA 2020 flow diagram by Page et al. [25].

Of the 82 reports sought for retrieval, 7 could not be retrieved, and 75 full‐text reports were assessed for eligibility. We excluded 56 reports at the full‐text stage: technical or information‐transfer communication focus without relational dimensions (*n* = 21), outside the healthcare context (*n* = 12), and insufficient conceptual detail or no communication dynamics (*n* = 23). A total of 19 studies met all inclusion criteria and were included in the concept analysis (see Figure 1).

The study selection process followed PRISMA 2020 reporting guidelines [25]. The database search yielded 4317 records: PubMed (*n* = 3024), Scopus (*n* = 1212), Web of Science (*n* = 27), and APA PsycNet (*n* = 54). Following the removal of 12 duplicates, 4305 records were screened by title and abstract against the criteria, with 4223 excluded at this stage.

## 6. Study Selection Process

The inclusion and exclusion criteria (see Table 1) were guided by PRISMA 2020 reporting standards [25] and Walker & Avant’s [23] concept analysis framework.

**TABLE 1:** Inclusion and exclusion criteria.

Criterion	Inclusion	Exclusion
Conceptual relevance	Studies addressing relational communication, communication competence, patient‐centered communication, or relational leadership in healthcare contexts, with explicit discussion of relational communication processes.	Studies focused solely on technical or information‐transfer communication without relational dimensions.
Population/context	Healthcare professionals, patients, families, or interdisciplinary teams in clinical, organizational, nursing management, or leadership development contexts.	Studies conducted outside healthcare contexts.
Language	English, peer‐reviewed.	Non‐English publications, conference abstracts, dissertations, editorials, commentaries, or non–peer‐reviewed sources.
Timeframe	January 2000–December 2024. Pre‐2000 foundational theoretical works were included where directly relevant, determined by two‐reviewer consensus and documented in the concept matrix.	Publications prior to 2000, except for foundational works as noted.
Source type	Empirical studies, theoretical papers, conceptual analyses, and prior concept analyses contribute conceptual insight into relational communication processes.	Studies focused solely on intervention outcomes without addressing communication dynamics.
Quality	Studies providing explicit discussion of communication processes, theoretical framing, or conceptual definitions relevant to RCC, assessed through full‐text review by two independent reviewers.	Studies lacking sufficient conceptual detail to support analytical synthesis.

### 6.1. Analytical Framework

Walker and Avant’s [23] eight‐step concept analysis framework was applied to examine RCC within nursing leadership and healthcare communication literature. Each step is described below with explicit linkage to the corresponding results.

#### 6.1.1. Step 1: Concept Identification

RCC was selected as the focal concept based on its conceptual fragmentation across nursing, leadership, and communication literature, as outlined in the Background. The working definition is refined through the analytical steps that follow, with the final conceptual definition presented in the Discussion. This provisional formulation served only to orient concept selection and was not used as a coding framework; the defining attributes were derived inductively from the included literature rather than mapped onto the working definition, thereby reducing the risk of circular reasoning.

#### 6.1.2. Step 2: Aim of the Analysis

The aims and objectives of this analysis are stated and are not repeated here.

#### 6.1.3. Step 3: Identification of Uses of the Concept

The included literature was examined to identify how RCC and related constructs are defined and used across healthcare communication and nursing scholarship [19, 21]. Conceptual definitions, theoretical frameworks, and communication processes were extracted and entered into a concept matrix that recorded study characteristics, conceptual definitions, theoretical frameworks, communication behaviors, and conceptual elements relevant to each analytical step. This process mapped the breadth and variation in how RCC has been conceptualized across the included sources (see Table 2).

**TABLE 2:** Summary of included studies.

Author (year)	Study design	Sample/data source (n)	Study origin/setting	Context	Theoretical/conceptual framework	Key conceptual focus	Concept elements identified	Contribution to relational communication competence
Asgari et al. [21]	Concept analysis (Walker and Avant method)	Literature sources identified through database search (no human sample) *n* = 40	Iran	Nursing leadership Healthcare management	Walker and Avant concept analysis framework	Communication competency of nurse managers	Communication skills, emotional capacity, interactive function ability, adapting to a situation, and communication knowledge	Identifies key attributes, antecedents, and consequences of communication competence in nurse leadership, informing conceptual development of relational communication competence Improved interpersonal relations, organizational effectiveness, job satisfaction, and reduced turnover
Hesse and Rauscher [8]	Cross‐sectional survey study Quantitative	*n* = 299 adult healthcare consumers reporting perceptions of doctor interactions (MTurk sample)	United States	Doctor–patient communication Healthcare	Affection exchange theory (AET)	Affectionate relational communication in healthcare	Affectionate communication, affection deprivation, trust, perceived communication competence, openness, satisfaction, adherence	Demonstrates that relational communication behaviors (affectionate communication) influence patient perceptions, trust, communication openness, satisfaction, and adherence in healthcare relationships The study highlights the importance of affectionate communication in enhancing patient perceptions and outcomes in healthcare settings, linking it to relational communication competence.
Kemp et al. [15]	Grounded theory qualitative study Longitudinal Study	*n* = 50 assisted living residents and 169 care convoy members (91 informal caregivers, 49 assisted living staff, 29 external healthcare providers)	United States	Assisted living communities (8 facilities) Healthcare delivery and communication processes in assisted living	Grounded theory approach; communicative competence concept derived from sociolinguistic theory (Hymes)	Collective communicative competence in recognizing and responding to resident health status changes	Identifying health changes; assessing the significance of health changes; responding to changes; informing, consulting, and collaborating among care partners; collective communicative competence	Demonstrates how communicative competence among residents and care partners shapes healthcare coordination and outcomes in long‐term care networks Enhanced understanding of communication processes around health changes in assisted living settings.
Kettunen et al. [34]	Qualitative discourse analysis (conversation analysis)	*N* = 19 nurses and 38 patients; 38 video‐recorded counseling sessions	Finland, hospital wards	Nurse–patient counseling interactions	Conversation analysis of interpersonal communication and power	Power dynamics in nurse–patient communication	Questioning, interruptions, disclosures, negotiation of interaction, asymmetrical power relations	Demonstrates how relational communication patterns shape participation and influence interactional control in healthcare encounters
Mahon and Nicotera [17]	Exploratory quantitative survey study	*n* = 57 practicing nurses (snowball sample; various specialties)	United States; inpatient and outpatient nursing settings	Workplace communication during shift‐change conflict	Communication Competence Theory; Conflict Communication Theory Health Communication Theory	Conflict communication competence in nursing	Constructive strategies (direct request, explanation, and cooperation) and destructive strategies (hostility, punishment, and avoidance)	Demonstrates how communication competence influences how nurses manage interpersonal conflict and workplace communication dynamics Nurses tend to prefer constructive communication strategies over destructive ones in conflict situations.
Nussbaum & Fisher [14]	Conceptual theoretical article; communication model development	Literature‐based conceptual model (no empirical sample)	Health communication scholarship; geriatric healthcare systems	Communication in geriatric medicine and interdisciplinary care	Communication Competence Theory; intergenerational communication; multidisciplinary health communication Communication accommodation theory	Communication competence in geriatric healthcare systems	Interdisciplinary communication, intergenerational communication, communication competence, collaborative healthcare communication Interdependence of health professionals, organizations, and social networks	Proposes a communication model placing communication competence at the center of coordinated geriatric healthcare among professionals, organizations, patients, and families
Omilion‐Hodges & Swords [30]	Qualitative study (semistructured interviews; grounded theory analysis)	*n* = 24 palliative care clinicians (22 physicians, 2 nurse practitioners)	United States; Circle of Life award‐winning palliative care programs	Palliative care leadership and clinician–patient communication	Mindfulness theory; mindful communication	Mindful communication practices in palliative care leadership	Audience awareness, active listening, authentic communication, relational presence, role awareness	Identifies relational communication practices that support empathetic engagement, trust development, and patient‐centered communication in interdisciplinary healthcare teams Enhances clinician–patient relationships and improves care delivery by promoting mindful communication practices.
Peltola et al. [31]	Qualitative methodological study using critical incident technique (CIT); literature review + empirical qualitative study	*n* = 41 patients with type 2 diabetes (open survey *n* = 16; interviews *n* = 25); 67 communication incidents analyzed	Finland; healthcare communication research	Professional–patient communication in diabetes (type 2) care	Critical incident technique interpersonal communication theory; relational dialectics theory	Critical communication incidents shaping professional–patient interaction and health‐related outcomes	Positive and negative communication experiences; relational interaction processes; communication behaviors facilitating or hindering care outcomes	Demonstrates how critical communication incidents reveal relational communication processes that influence patient understanding, engagement, and health‐related outcomes in healthcare interactions. Enhanced understanding of interpersonal communication experiences in diabetes management
Pretorius [35] 2023?	Quantitative training evaluation (pre–post design) using a questionnaire and language competence tests	*n* = 68 overseas educated nurses (OENs) participating in a pre–post communication training evaluation	U.K. hospitals	Communication training for internationally educated nurses	Communication Accommodation Theory (CAT); communicative competence frameworks	Communicative competence in multilingual nurse–patient communication Self‐perceived vs. actual communicative competence	Task‐focused communication competence; rapport‐building competence; self‐perceived vs. actual competence; accommodative communication behaviors	Demonstrates how communicative competence involves both informational and relational communication processes, highlighting accommodation, rapport‐building, and adaptive communication as components of relational communication competence Improved self‐perceived competence and alignment with actual competence post‐training
Pretorius [32] 2017	Qualitative observational study with interviews and discourse analysis	*n* = 249 participants observed in nurse–patient interactions (including 79 nursing staff across ranks); 15 nurses interviewed	Two hospitals in Bloemfontein, South Africa	Language‐discordant nurse–patient communication	Communication Accommodation Theory (CAT) integrated with patient‐centered communication literature	Effects of linguistic competence and accommodative behavior in nurse–patient interaction Nurse–patient communication and language barriers	Accommodation strategies (convergence and divergence), task‐focused communication, rapport‐building communication, affective communication, perception of motives, evaluative responses Linguistic competence issues (phonetics and lexicon)	Demonstrates how relational communication competence emerges through accommodative communication behaviors, rapport‐building, and affective responsiveness in multilingual healthcare interactions Understanding the impact of linguistic competence on nurse–patient interactions and informing training needs for relational communication competence
Robins et al. [26]	Qualitative communication analysis (audio‐recorded consultations with coding)	263 recorded physician–patient consultations involving 33 primary care physicians and 259 adult patients	Eight community‐based primary care clinics, Washington State, United States	Physician–patient communication in primary care encounters	Patient‐centered communication; transparency in clinical interaction	Transparent physician communication	Process transparency (agenda, framing, metacommentary, exam explanation, orchestration) and content transparency (demystification, diagnostic rationale, treatment rationale, opinion sharing)	Identifies communication transparency behaviors that enhance understanding, trust, and collaboration in clinical encounters
Rosa et al. [33]	Qualitative thematic analysis of open‐ended survey responses	*n* = 152 interdisciplinary clinicians (73 nurses, 54 social workers, 25 chaplains)	United States (participants recruited through National Cancer Institute Interdisciplinary Communication Curriculum program)	Palliative care and end‐of‐life communication with racially and culturally minoritized patients with cancer	Person‐centered communication and culturally inclusive palliative care frameworks	Person‐centered and culturally sensitive communication in end‐of‐life care	Person‐centered communication, authentic care, trust building, culturally sensitive dialog, addressing racism, empathic communication	Demonstrates relational communication processes required to build trust, empathy, and culturally responsive interaction in serious illness communication
Ross & Castle Bell [27]	Qualitative study using semistructured interviews and thematic analysis	*n* = 13 transgender individuals (self‐identified trans community members aged ≥ 18)	United States (participants from California, North Carolina, New York, Texas, Washington)	Transgender patient–practitioner communication in healthcare encounters	Culture‐Centered Approach to Health Communication (Dutta, 2007)	Improving patient–practitioner communication for transgender patients	Respectful communication, preferred pronoun use, acknowledgment of identity, communication sensitivity, relational respect, inclusive communication environments	Demonstrates how culturally responsive and relationally sensitive communication behaviors improve healthcare interactions and trust in marginalized patient populations Improving communication practices and healthcare experiences for transgender individuals
Sabee et al. [7]	Mixed‐method communication analysis (qualitative + quantitative coding)	(55 audio‐recorded medical visits = Phase 1; 50 analyzed samples = Phase 2) 50 recorded medical consultations; 52 patients with Type 2 diabetes and 52 physicians (30 primary care physicians, 22 endocrinologists); 602 interaction units analyzed	United States—clinical consultations	Physician–patient communication in chronic illness care	Interactional Sensitivity framework integrated with patient‐centered communication (PCC) model	Interactional sensitivity in patient‐centered communication and PCC framework	Exchange of information; fostering relationships; managing uncertainty; meta‐communication; decision‐making processes	Demonstrates how relational communication behaviors emerge through interactional sensitivity between healthcare providers and patients Developed the Process for Interactional Sensitivity Coding in Healthcare (PISCH) to evaluate PCC in an interactional context focused on chronic illness.
Step et al. [5]	Observational communication study using coded clinical consultations Cross‐sectional observational study (secondary analysis of RCT data)	180 audio‐recorded oncology consultations; breast cancer patients (Stages I–III) interacting with 24 oncologists	United States—oncology clinics (14 practices)	Oncologist–patient treatment decision consultations Communication in patient‐centered care	Relational Communication Theory within patient‐centered communication framework	Relational communication and patient communication involvement in medical decision‐making	Confirming messages, relational immediacy, patient involvement, communication climate Instrumental communication, relational communication, and decision regret	Demonstrates how clinicians’ relational communication behaviors increase patient communication involvement and influence decision outcomes Positive oncologist communicative behaviors enhance patient involvement and reduce decision regret
Thompson et al. [28]	Pre–post consultation survey study Mixed methods (prospective, observational, pre–post (within‐subject) study using multilevel (hierarchical) correlational analysis)	*n* = 200 chronic pain patients and 6 physicians	United States—Midwestern Medical Institute	Chronic pain consultation (patient–physician interaction)	Uncertainty Management Theory + Medical Communication Competence	Informational and socio‐emotional communication competence in clinical consultations	Informational communication, socio‐emotional communication, trust, uncertainty reduction, patient self‐efficacy	Demonstrates how relational communication competence between physicians and patients influences uncertainty management and patient outcomes Patients’ uncertainty reduction, emotional appraisals, and pain self‐efficacy
Tobiano et al. [29]	Observational study (ethnographic approach)	*n* = 28 nurse–patient dyads; 108 observed encounters	Australia—public hospital medical wards	Nurse–patient interaction during inpatient care	Patient participation framework (Eldh et al.) and patient‐centered care theory	Communication activities promoting patient participation	Dialog, knowledge sharing, planning participation, self‐care engagement, relational interaction	Demonstrates how relational nurse–patient communication supports patient participation and partnership in care Identifies barriers and facilitators to patient participation through relational communication.
Wanzer et al. [6]	Cross‐sectional survey study and a quantitative correlational survey study (observational design)	*n* = 195 parents of hospitalized pediatric patients	United Stateslarge children’s hospital	Parent perceptions of physicians, nurses, and staff communication	Patient‐centered communication (PCC) model + Uncertainty Reduction Theory	Communication behaviors influencing satisfaction with care	Empathy, listening, clarity, introductions, immediacy, humor	Demonstrates how relational communication behaviors shape patient satisfaction and provider–patient relationships
Wittenberg‐Lyles et al. [18]	Qualitative observational communication analysis	*n* = 81 hospice interdisciplinary team discussions	U.S. hospice teams	Interdisciplinary team communication	Relational control theory	Relational communication in healthcare teams	Relational control messages (one‐up, one‐down, one‐across), interactional dominance patterns, relational communication dynamics	Demonstrates how relational control messages and interactional dominance patterns shape communication dynamics and influence collaboration within interdisciplinary healthcare teams.

#### 6.1.4. Step 4: Defining Attributes

Extracted conceptual elements were compared iteratively across the included studies to identify characteristics recurring consistently and distinctively across the literature. Attributes were considered defining when they appeared across multiple independent sources and were confirmed through two‐reviewer consensus as representing a core characteristic of RCC rather than a peripheral or context‐specific feature. Where reviewers disagreed, discrepancies were discussed until consensus was reached; no third reviewer was required. Three defining attributes emerged: relational sensitivity, effective information exchange, and collaborative engagement (see Table 3).

**TABLE 3:** Defining attributes of relational communication competence (RCC).

Attribute	Definition	Illustrative examples from the included articles
Relational sensitivity	The ability to perceive, interpret, and respond adaptively to the emotional, interpersonal, cultural, and contextual dimensions of healthcare interactions.	Clinicians demonstrate empathy, attentiveness, and responsiveness during interactions, fostering trust and relational connection between healthcare professionals and patients [6, 7]. Communication that acknowledges patients’ cultural identities, experiences, and perspectives also strengthens respectful healthcare relationships (Ross & Bell, 2017; [33]).
Effective information exchange	The ability to communicate clinical information clearly, transparently, and responsively while fostering shared understanding between healthcare providers, patients, and families.	Transparent explanations of diagnoses, procedures, and treatment plans help patients understand their health conditions and support informed decision‐making [26]. Integrating informational clarity with socio‐emotional responsiveness during clinical consultations reduces uncertainty, supports understanding, and improves patient outcomes [14, 15, 28].
Collaborative engagement	The ability to communicate in ways that promote relational dialog, participation, shared decision‐making, and coordinated interaction among patients, families, and healthcare teams.	Relational communication behaviors such as confirming messages, openness, and dialog encourage patient participation in treatment decisions and healthcare discussions [5]. Dialog and information sharing between nurses and patients support participation in care processes [29], while communication patterns among healthcare professionals influence collaboration and decision‐making within interdisciplinary teams [18, 34].

#### 6.1.5. Step 5: Model Case

A model case was constructed as a hypothetical but clinically realistic scenario illustrating the simultaneous presence of all three defining attributes within an interdisciplinary older‐adult care interaction. The case was reviewed for conceptual accuracy against the defining attributes prior to inclusion in the manuscript (see Table 4).

**TABLE 4:** Model case of relational communication competence.

Attribute demonstrated	Model case illustration
Relational sensitivity	Maria, a nurse manager in a long‐term care unit, meets with the interdisciplinary team to discuss the care of an older patient experiencing worsening pain and emotional distress. As the bedside nurse describes the patient’s concerns, Maria listens attentively and acknowledges the patient’s anxiety, demonstrating empathy and respect for both the patient and the nurse’s observations.
Effective information exchange	Continuing the discussion, Maria clearly explains the patient’s recent assessment findings, symptoms, and possible treatment options to the interdisciplinary team. She ensures that the information is communicated in language that is understandable to both healthcare professionals and the patient’s family members.
Collaborative engagement	Maria then invites the physician, social worker, and family members to share their perspectives and encourages open discussion about the patient’s care plan. Through this collaborative dialog, the team develops a shared and patient‐centered approach to managing the patient’s symptoms.

#### 6.1.6. Step 6: Borderline, Related, and Contrary Cases

Additional cases were developed to clarify the conceptual boundaries of RCC ([23], pp. 168–171). A borderline case demonstrates the partial presence of the defining attributes; a related case illustrates a communication process that resembles but does not fully represent RCC; and a contrary case demonstrates the complete absence of all defining attributes, distinguishing RCC from conceptually related but distinct communication constructs (see Table 5).

**TABLE 5:** Borderline, related, and contrary cases of relational communication competence.

Case type	Description	Defining attributes present
Borderline case	A nurse manager communicates a patient’s treatment update clearly to the healthcare team, ensuring that clinical information is accurately conveyed. However, the manager does not acknowledge the patient’s emotional concerns or invite input from other team members during the discussion. Communication occurs, but the interaction remains task‐focused and lacks relational sensitivity and collaborative dialog.	Effective information exchange only
Related case	A clinician employs therapeutic communication during a patient encounter, demonstrating empathic presence, active listening, and emotional support. The interaction does not include a clear exchange of clinical information or an invitation for shared decision‐making. The encounter is relationally attentive but focused on emotional support within the individual clinician–patient relationship, without addressing the informational or collaborative dimensions that RCC requires [21, 26].	Relational sensitivity only
Contrary case	A nurse manager abruptly informs staff that a patient’s care plan has changed without explaining the reasons for the change. Questions from staff are dismissed, and concerns raised by the patient’s family are ignored. The interaction is directive and dismissive, demonstrating no relational sensitivity, clear information exchange, or collaborative engagement.	None of the defining attributes are present

#### 6.1.7. Step 7: Antecedents and Consequences

The included literature was examined to identify conditions preceding the occurrence of RCC and outcomes associated with its presence in healthcare interactions. Antecedents and consequences were extracted independently by two reviewers and organized inductively into thematic categories through iterative comparison across sources. Discrepancies were resolved through discussion until consensus was reached (see Table 6).

**TABLE 6:** Antecedents and consequences of relational communication competence.

Antecedents are conditions or events occurring prior to the concept that enable or influence relational communication competence.			Consequences are outcomes that emerge after relational communication competence is demonstrated in practice.		
Antecedent	Definition (what it is)	Illustrative examples (practice)	Consequence	Definition (what it is)	Illustrative examples (practice)
Communication knowledge and skill preparation	Foundational knowledge of communication processes, along with the interpersonal skills needed to listen, explain, question, clarify, and respond appropriately in healthcare interactions.	A nurse leader has prior preparation in communication competence and uses questioning, explanation, and clarification effectively during care discussions with staff, patients, and families.	Enhanced therapeutic relationships	Development of stronger, trust‐based relationships between healthcare professionals and patients through empathic and responsive communication.	A nurse who listens attentively and acknowledges patient concerns fosters a sense of trust, allowing patients to feel respected and supported during care discussions.
Awareness of emotional, relational, and cultural needs	Recognition that healthcare communication involves not only clinical information but also emotional experience, relational meaning, identity, and cultural context.	A clinician recognizes that a patient’s fear, mistrust, cultural identity, or emotional distress will shape the interaction and approaches the conversation with sensitivity rather than treating it as a purely technical exchange.	Improved patient engagement and participation	Increased involvement of patients in discussions, decision‐making, and care planning processes.	Patients actively ask questions, share concerns, and participate in decisions about treatment options during consultations or care meetings.
Opportunity for dialog and mutual information sharing	The presence of interactional space in which patients, families, and professionals can express concerns, ask questions, share knowledge, and verify understanding.	During a consultation or team meeting, patients are encouraged to ask questions, family members are invited to share concerns, and staff can discuss care issues rather than simply receiving one‐way instructions.	Greater patient satisfaction and perceived quality of care	Positive patient perceptions of healthcare interactions and overall care experience result from respectful and responsive communication.	Patients report feeling understood and valued when clinicians communicate clearly and respond to emotional and informational needs.
Supportive interdisciplinary and organizational communication climate	A team or organizational environment that supports open communication, role contribution, shared discussion, and collaboration across disciplines.	In interdisciplinary meetings, nurses, physicians, social workers, and other professionals are able to contribute their perspectives without communication being restricted to a single dominant voice.	Improved interdisciplinary collaboration	More effective teamwork and coordination among healthcare professionals through open dialog and relational communication practices.	Nurses, physicians, and other healthcare professionals engage in collaborative discussions during multidisciplinary meetings to coordinate patient care plans.
Organizational and clinical structures enabling participation and shared decision‐making	Organizational or clinical arrangements that create opportunities for patients, families, and healthcare professionals to participate in discussions, decision‐making, and care planning.	A nurse invites a patient to discuss treatment preferences, or a team leader actively involves staff and family members in planning the next steps of care rather than announcing decisions unilaterally.	Improved shared clinical decision‐making and care coordination	More coherent and effective clinical decisions result from shared understanding and collaborative communication among healthcare professionals and patients.	Care teams develop coordinated care plans through discussion that integrates clinical information, patient preferences, and professional perspectives.
Communication training and professional learning supports	Educational or professional learning experiences that strengthen communication competence and support the development of relational, patient‐centered, and collaborative communication practices.	Nurses or clinicians participate in communication training focused on rapport‐building, accommodation, patient‐centered dialog, uncertainty management, or interdisciplinary teamwork.	Reduction of uncertainty in healthcare interactions	Decreased ambiguity and confusion for patients and professionals when information and relational communication are effectively integrated.	Clear explanations of diagnoses and treatment plans help patients understand their condition and feel more confident about care decisions.

#### 6.1.8. Step 8: Empirical Referents

Observable communication behaviors reflecting each defining attribute were identified from the included literature [5, 7, 26]. Empirical referents were selected based on their capacity to render each defining attribute measurable and observable in clinical and organizational nursing practice (see Table 7).

**TABLE 7:** Empirical referents of relational communication competence.

Defining attribute	Empirical referents (observable indicators)
Relational sensitivity	Empathic listening, acknowledgment of patient emotions, respectful language, and culturally responsive communication
Effective information exchange	Clear explanation of diagnoses and treatment plans, use of understandable language, and verification of patient understanding
Collaborative engagement	Invitation for patient or team input, shared decision‐making dialog, open discussion during interdisciplinary meetings

### 6.2. Data Extraction and Conceptual Synthesis

Two researchers with backgrounds in nursing leadership and healthcare communication conducted data extraction and conceptual synthesis. Each of the 19 included studies was reviewed in full and entered into the concept matrix, enabling structured comparison of conceptual elements across sources. Following Walker and Avant’s [23] Steps 3 and 4, conceptual elements were identified and labeled across the included sources, then compared iteratively to identify recurring patterns and conceptual similarities. Related concepts and communication behaviors were clustered inductively into broader categories representing the defining attributes of RCC, derived directly from patterns observed across the included literature rather than mapped against the theoretical frameworks.

To illustrate this process, empathic listening described by Sabee et al. [7], culturally responsive interaction reported by Ross and Castle Bell [27], and attentiveness to patients’ emotional cues noted by Wanzer et al. [6] were initially coded as discrete elements; on iterative comparison, their shared conceptual core was recognized, and they were consolidated into the single defining attribute of relational sensitivity. Similarly, transparent clinical explanation reported by Robins et al. [26], verification of shared understanding described by Thompson et al. [28], and uncertainty reduction discussed by Kemp et al. [15] were grouped under effective information exchange because they shared a common concern with clarity, transparency, and mutual understanding. In the same way, confirming messages described by Step et al. [5], patient participation reported by Tobiano et al. [29], and interdisciplinary dialog examined by Wittenberg‐Lyles et al. [18] were synthesized as collaborative engagement because they reflected participation, shared decision‐making, and coordinated dialog across patients, families, and healthcare professionals.

We maintained an audit trail throughout to document interpretive decisions at each analytical step. The two researchers resolved disagreements through discussion until reaching consensus, supporting transparency and traceability of the conceptual relationships presented (Tables 1, 2, 3, 4, 5, 6, 7). No new conceptual attributes emerged from the final studies reviewed, indicating sufficient conceptual coverage of the literature to support the defining attributes identified in this analysis.

### 6.3. Rigor and Trustworthiness

We addressed analytical transparency through clear documentation of the search strategy, screening, and study selection, guided by a PRISMA‐adapted flow diagram (see Figure 1) and consistent inclusion and exclusion criteria. Applying defining attributes across studies and cases using a structured concept matrix supported conceptual consistency. Two researchers conducted the analysis and resolved differences through discussion. We maintained an audit trail as a shared record of analytical decisions throughout the Walker and Avant process [23]. Regular team discussions reviewed attribute identification, case development, and the identification of antecedents and consequences. The researchers’ professional backgrounds in nursing leadership and healthcare communication represent a reflexive consideration: These backgrounds may have oriented attribute identification toward these domains. This reflexive risk was mitigated through independent attribute identification by each researcher before any consensus discussion, ensuring initial coding decisions were not influenced by each other’s professional frameworks. These procedures support the overall rigor of the analysis [24].

## 7. Results

### 7.1. Summary of Included Studies

A total of 19 studies met the inclusion criteria and were included in the concept analysis, published between 2002 and 2024. The literature represented four methodological categories: qualitative studies (*n* = 7; [15, 27, 29–33]), observational communication analyses (*n* = 5; [5, 7, 18, 26, 34]), quantitative survey studies (*n* = 5; [6, 8, 17, 28, 35]), and conceptual or theoretical articles (*n* = 2; [14, 21]). Together, these four categories account for all 19 included studies.

Studies originated from six countries: the United States (*n* = 13), Finland (*n* = 2), South Africa (*n* = 1), Australia (*n* = 1), Iran (*n* = 1), and the United Kingdom (*n* = 1). The predominance of North American studies limits the geographic breadth of the conceptual findings, and the international applicability of RCC should be interpreted accordingly. Healthcare settings included nurse–patient counseling [34], physician–patient consultations in primary and oncology care [5, 26], interdisciplinary teams [18], palliative care [30, 33], long‐term care and chronic illness management [7, 15, 31], and nursing workplace communication [17]. This range of settings supports the applicability of RCC across varied clinical and organizational nursing contexts.

Across the literature, RCC was examined through varied theoretical perspectives, including affection exchange theory [8], communication accommodation theory [35], interactional sensitivity [7], relational communication theory [5], and communication competence frameworks in nursing and geriatric care [14, 21]. This theoretical variation reflects the terminological fragmentation that justified the concept analysis approach. Despite this variation, the included studies consistently identified empathy, relational responsiveness, transparency in information exchange, and collaborative dialog as central communication processes aligning with the three defining attributes of relational sensitivity, effective information exchange, and collaborative engagement identified in this analysis (see Table 2).

### 7.2. Identification of Uses of the Concept

Although the specific term “*relational communication competence”* rarely appears explicitly in the healthcare literature ([19, 21], p. 7), several surrogate terms and related constructs describe similar communication processes. Surrogate terms are different words used to describe the same concept; related concepts share some but not all defining attributes of RCC ([23], p. 157).

Surrogate terms including patient‐centered communication (*n* = 8 studies; [5–8, 26, 28, 29, 33]), communication competence (*n* = 5; [14, 15, 17, 21, 35]), and communicative competence (*n* = 3; [7, 31, 34]) consistently emphasized relational responsiveness, empathic listening, and contextually appropriate communication as central to effective healthcare interactions.

Related concepts and constructs sharing some but not all attributes of RCC include communication accommodation [35], interactional sensitivity [7], mindful communication [30], relational communication theory [5], and relational control theory [18]. These constructs highlight adaptation to linguistic and cultural needs, responsiveness to emotional and relational cues, genuine relational engagement, and communication behaviors, including confirmation, immediacy, and transparency in information exchange that influence patient participation and understanding during clinical encounters.

Across these uses, three recurring conceptual patterns emerged: relational responsiveness, transparent information exchange, and collaborative dialog directly informing the defining attributes of relational sensitivity, effective information exchange, and collaborative engagement identified in Step 4. The consistency of these patterns across varied theoretical traditions strengthens the analytical basis for the three defining attributes (see Table 3).

### 7.3. Defining Attributes

Defining attributes are the core characteristics that repeatedly appear in the literature and distinguish a concept from related constructs ([23], p. 157). Through iterative comparison across the included studies and two‐reviewer consensus, three defining attributes of RCC were identified: relational sensitivity, effective information exchange, and collaborative engagement (see Table 3). Observable indicators of each attribute are presented in Table 7. These attributes are analytically distinct but functionally interdependent; their simultaneous presence characterizes RCC within a healthcare interaction, as illustrated in the model case and contrary case.

#### 7.3.1. Relational Sensitivity

This attribute denotes the capacity to perceive, interpret, and respond appropriately to the emotional, interpersonal, cultural, and contextual dimensions of healthcare communication (*n* = 12 studies). Empathy and relational responsiveness foster trust and strengthen therapeutic relationships [6, 7], while culturally responsive communication strengthens respectful relationships across diverse patient populations [27, 33]. Unlike interactional sensitivity, relational sensitivity encompasses cultural and contextual dimensions beyond moment‐to‐moment clinical responsiveness. Without it, communication may be technically accurate but relationally inadequate, and RCC cannot be said to be present. Within nursing leadership, relational sensitivity shapes how nurse leaders create environments in which staff and patients feel heard and respected.

#### 7.3.2. Effective Information Exchange

This attribute denotes the capacity to communicate clinical information clearly and transparently while ensuring shared understanding among professionals, patients, and families (*n* = 11 studies). This attribute distinguishes RCC from purely technical communication competence by requiring relational responsiveness alongside informational clarity. Transparent clinical explanations improve patient understanding and informed decision‐making [26, 28], while integrating relational and informational dimensions reduces uncertainty and improves patient outcomes [14, 15]. Without effective information exchange, relational awareness alone cannot support safe and coordinated care, and RCC cannot be said to be present.

#### 7.3.3. Collaborative Engagement

This attribute encompasses communication practices promoting dialog, participation, and shared decision‐making among patients, families, and healthcare professionals (*n* = 12 studies). Unlike patient‐centered communication, it operates at both individual patient and interdisciplinary team levels. Confirming messages, openness, and supportive dialog encourage patient involvement in treatment discussions [5, 29], while relational communication patterns facilitate coordinated decision‐making within teams [18, 34] and open communication across disciplines in nursing leadership contexts [17]. Without collaborative engagement, communication remains directive rather than participatory, and RCC cannot be said to be present.

Relational sensitivity is the most foundational attribute; it enables recognition of emotional and contextual cues that shape how information is communicated and whether dialog is possible. Effective information exchange ensures clinical information is clearly understood, and collaborative engagement promotes shared participation in care decisions and team coordination [5, 7].

### 7.4. Model Case

A model case illustrates a situation in which all defining attributes are fully and simultaneously present, distinguishing it from the borderline case ([23], p. 163; see Table 4). This case embodies the conceptual definition of RCC as the capacity to engage in communication that is empathic, contextually responsive, and relationship‐sustaining in professional and care contexts.

Maria, a nurse manager in a long‐term care unit, facilitates an interdisciplinary team meeting for an older patient experiencing worsening pain and emotional distress compounded by early cognitive decline. The patient’s daughter attends as surrogate decision‐maker. Maria invites the bedside nurse to share observations before medical input is sought, creating space for nursing perspectives and modeling relational communication for the team [1].

Maria presents assessment findings in clear, accessible language, adapting her communication to ensure the patient’s cognitive status does not exclude her from the discussion. She invites questions from the team and family, verifying shared understanding before proceeding, integrating relational responsiveness with informational clarity ([19], p. 7).

Maria facilitates dialog among the physician, social worker, and family, ensuring all perspectives contribute to a jointly developed person‐centered care plan [13]. The plan is documented and communicated to all team members after the meeting. The interaction is not friction‐free: The physician initially redirects the discussion toward discharge timelines, and the patient’s fatigue limits her verbal participation. Maria navigates both without abandoning the relational framework, returning to the patient’s expressed concerns and recalibrating the pace of information exchange accordingly. Relational sensitivity, effective information exchange, and collaborative engagement are each fully present, confirmed through the observable indicators in Table 7, and together constitute RCC within this interaction.

### 7.5. Borderline, Related, and Contrary Cases

Three additional cases clarify the conceptual boundaries of RCC ([23], pp. 168–171; see Table 5).

#### 7.5.1. Borderline Case

A nurse manager facilitates a family meeting for an older patient, acknowledging family concerns and inviting staff and family to share perspectives, demonstrating relational sensitivity and collaborative engagement. However, clinical information is communicated using medical terminology that the patient and family misunderstand, and shared understanding is never verified. Effective information exchange is absent. This reflects a common clinical reality: Even attentive communicators can default to technical language under time pressure or professional habit, compromising clarity despite genuine relational intent ([17]; see Table 7). All three attributes must be present simultaneously for RCC to be characterized; the absence of any one of them means RCC cannot be said to be present in this interaction.

#### 7.5.2. Related Case

Therapeutic communication, a nursing construct emphasizing empathic presence and patient support, shares relational sensitivity with RCC. However, it is concerned primarily with the individual clinician–patient relationship and does not address effective information exchange or collaborative engagement across interdisciplinary teams and care decisions. It therefore represents a conceptually distinct construct that overlaps with but does not constitute RCC [21, 26].

#### 7.5.3. Contrary Case

A nurse manager informs staff that an older patient’s care plan has changed without explanation. Key antecedents, including organizational communication support and opportunities for dialog, are absent (see Table 6). Questions are dismissed, and family concerns are ignored despite the patient’s cognitive decline. No defining attribute is present, and the consequences are direct: Patient safety is compromised, team relationships deteriorate, and the family is excluded from care decisions [14, 18].

## 8. Discussion

Overall, the three defining attributes suggest that RCC in nursing leadership cannot be fully explained by any single adjacent construct, although it clearly shares conceptual territory with several of them. Relational sensitivity, effective information exchange, and collaborative engagement each appear separately across prior frameworks; however, existing frameworks appear not to have consistently specified their simultaneous co‐occurrence as the condition under which RCC is present.

The refined conceptual definition that emerges from this analysis is as follows: RCC is the capacity to engage in healthcare communication that integrates empathic responsiveness to relational and contextual cues, clear and transparent exchange of clinical information, and facilitation of collaborative dialog among patients, families, and healthcare professionals. This definition is integrative rather than merely additive. It addresses fragmentation in the literature, where relational communication has been theorized inconsistently as an interpersonal skill in some accounts, a leadership process in others, and a person‐centered care value in still others without a unified account of how these dimensions operate together in nursing leadership and management contexts [1, 21]. It extends conventional communication competence frameworks, which have predominantly emphasized appropriate and effective message transmission ([19], p. 7), by showing that communication in nursing leadership also requires relational awareness and collaborative involvement in addition to informational clarity [36].

The findings are consistent with and build on all three theoretical frameworks underpinning this analysis. Relational Leadership Theory [20] positions leadership as emerging through mutual influence and trust, consistent with research in nursing leadership that shows how leader communication shapes the relational environments in which teams coordinate care [1]. Communication Competence Theory ([19], p. 7) defines competence as contextually appropriate and effective. The findings extend that concept by showing that in nursing leadership contexts, appropriateness requires cultural and emotional responsiveness, not merely situational fit. That is, competent communication in nursing is not simply a matter of adapting to the immediate interaction but requires sustained awareness of the relational, cultural, and emotional contexts that shape every clinical encounter. The Person‐Centered Practice Framework [13] places empathic engagement and collaborative partnership at the center of nursing practice. The findings ground these principles in specific, observable communication behaviors within clinical and leadership contexts [15, 37]. While skill‐based frameworks provide valuable tools for assessing discrete communication behaviors, they do not account for how a nurse leader’s communication shapes the psychological safety of the team or the degree to which patients feel genuinely heard, dimensions that lie outside individual behavioral performance ([19], p. 7; [17]).

Relational Leadership Theory [20] and Communication Competence Theory ([19], p. 7) share a relational orientation but operate at different levels of analysis: organizational and interpersonal, respectively. Their integration within RCC rests on the assumption that leadership processes and communicative competence are mutually constitutive at the level of clinical interaction, an assumption appropriate within nursing leadership contexts but requiring empirical testing before it can be adopted as axiomatic. RCC as a relational capability also presupposes conditions that are frequently absent in under‐resourced or high‐acuity settings. In acute care, time constraints may limit the relational attunement that relational sensitivity demands. Hierarchical team structures may constrain collaborative engagement even when individual willingness is present. The three attributes may also be differentially weighted across clinical specialties: Relational sensitivity may be most salient in palliative and long‐term care, while effective information exchange may be proportionally more critical in acute and critical care settings. These tensions between RCC as a conceptual ideal and the organizational realities of nursing leadership represent important parameters that implementation‐focused research must systematically address.

## 9. Conceptual Differentiation

RCC must be distinguished from related constructs that share some, but not all, of its defining attributes. Therapeutic communication emphasizes the empathic dimension of the clinician–patient relationship but does not treat effective information exchange as a distinct simultaneous attribute or address collaborative decision‐making across interdisciplinary teams [21, 26]. Patient‐centered communication functions primarily at the level of the individual clinical encounter; RCC extends to both patient care and organizational leadership, requiring simultaneous competence at the relational, informational, and collaborative levels [6, 7]. Communication competence in its foundational formulation ([19], p. 7) defines competence as contextually appropriate and goal‐effective behavior but does not integrate Relational Leadership Theory or person‐centered care values. Relational leadership theorizes leadership as emerging through trust and mutual influence, yet does not specify the communication behaviors through which this relational work is enacted. Interprofessional communication frameworks focus on coordination and information exchange across disciplines but do not consistently theorize the empathic and relational dimensions that RCC positions as foundational [17, 18]. RCC is therefore best understood as an integrative construct requiring the simultaneous presence of all three defining attributes. Table 8 summarizes these distinctions across relational, informational, and collaborative dimensions.

**TABLE 8:** Conceptual differentiation of RCC from related communication and leadership constructs.

Construct	Relational sensitivity	Effective information exchange	Collaborative engagement	Key distinction from RCC
RCC (this analysis)	Required	Required	Required	Defined by the simultaneous presence of all three attributes within a single interaction, spanning patient, team, and organizational levels.
Therapeutic communication	Central	Not a distinct attribute	Not addressed	Empathic clinician–patient focus does not treat information exchange as a distinct, simultaneous attribute or address interdisciplinary decision‐making [21, 26].
Communication competence (Spitzberg and Cupach)	Partial	Central	Partial	Defines competence as contextually appropriate and goal‐effective behavior; does not integrate relational leadership or person‐centered care values [19].
Patient‐centered communication	Central	Present	Partial	Operates at the level of the individual clinical encounter; does not extend to organizational leadership [6, 7].
Relational leadership	Central	Not specified	Present	Theorizes leadership as emerging through trust and mutual influence but does not specify the communicative behaviors that enact it [20].
Interprofessional communication	Not theorized	Central	Central	Focuses on coordination and information exchange across disciplines; does not theorize the empathic and relational dimensions RCC positions as foundational [17, 18].

Compared with Asgari et al. [21], whose concept analysis of nurse manager communication competence is the most closely related existing work, RCC offers a broader conceptualization. Asgari et al. identified communication skills, emotional capacity, and adaptive function as defining attributes, with an emphasis on the individual nurse manager. In contrast, this analysis reframes communication competence as a relational process spanning individual, team, and organizational levels, examining both what nurse leaders communicate and how relational, informational, and collaborative dimensions interact in healthcare [1, 18]. RCC is not a personal skill set but a relational capability situated within professional relationships and organizational contexts [20]. Whereas a skill set resides in the individual and can be trained in isolation, a relational capability emerges through interaction and depends on the quality of relationships and environments in which communication occurs [38]. This distinction is significant for nursing management, redirecting attention from isolated skill training to cultivating relational conditions that enable effective team communication.

The antecedents identified, including communication knowledge, relational and cultural awareness, opportunities for dialog, supportive organizational communication climates, and professional training [18, 35], suggest that RCC does not emerge spontaneously but develops only under sustained organizational conditions. Nurse managers are well positioned to shape these conditions within their teams, for example, by structuring meetings to embed deliberate relational communication practices, investing in culturally responsive communication training, and modeling empathic engagement in clinical interactions.

The consequences, including enhanced therapeutic relationships, improved patient engagement, stronger interdisciplinary collaboration, and reduced clinical uncertainty, indicate that RCC has important implications for care quality and team effectiveness [15]. The combination of antecedents and consequences suggests that communication improvement in nursing requires organizational investment, not only individual skill development.

The leadership relevance of RCC extends beyond individual clinical interactions. Relational sensitivity, when enacted consistently by nurse leaders, is associated with staff engagement and retention [1] and may help create conditions of psychological safety in which staff feel heard and supported [17]. Collaborative engagement aligns with transformational leadership behaviors, particularly facilitating shared decision‐making and strengthening team cohesion [37]. Effective information exchange supports patient safety culture by reducing clinical uncertainty and communication breakdowns linked to error [4]. Thus, these links position RCC as relevant not only to person‐centered care quality but also to the organizational infrastructures that sustain a competent and engaged nursing workforce.

The empirical referents identified, including empathic listening, acknowledgment of emotional concerns, transparent explanation of clinical information, verification of shared understanding, and collaborative dialog [7, 26], make each defining attribute observable in clinical and leadership practice. Their identification opens a practical path toward incorporating RCC into frameworks for nursing leadership competencies, performance evaluation, and nursing education. For instance, Asgari et al. [21] noted that empathic listening and verification of shared understanding could be incorporated as observable performance indicators within existing appraisal frameworks for nurse managers, addressing a gap where a clearly defined relational communication construct has been absent. While grounded in a predominantly North American evidence base, their alignment with international person‐centered care principles suggests broader applicability, though systematic cross‐cultural validation remains essential.

This analysis offers a conceptual account of RCC that remains underdeveloped in nursing leadership scholarship. It clarifies how relational, informational, and collaborative dimensions of communication may intersect in nursing leadership and person‐centered care, providing nurse educators, managers, and researchers with a shared conceptual language for developing, evaluating, and studying relational communication in nursing leadership ([19, 21], p. 7).

The primary contribution of this analysis is integrative in scope. Existing frameworks address relational leadership [20], communication competence ([19], p. 7), and person‐centered care [13, 22] as parallel bodies of scholarship that share a relational orientation but have developed largely in isolation. Few existing frameworks appear to have operationalized how these three domains intersect within a single healthcare interaction. RCC therefore offers, to our knowledge, an early unified conceptual definition specifying how this intersection is constituted, what antecedent conditions enable it, what outcomes are associated with it, and what observable behaviors may indicate its presence, while remaining open to overlap with adjacent relational and communicative constructs. The contribution is integrative but not merely additive: The simultaneous presence of all three attributes describes a relational capability that the source frameworks do not appear to fully articulate or measure in combination.

## 10. Limitations

Several limitations should be considered when interpreting the findings. First, 13 of 19 included studies originated from the United States, which limits transferability to healthcare systems characterized by different organizational structures, professional role boundaries, or cultural communication norms. The conceptualization of relational sensitivity, particularly its cultural and contextual dimensions, may manifest differently across healthcare systems with distinct communication norms, hierarchical structures, and patient‐professional relationship traditions. The international applicability of RCC therefore requires empirical validation before the framework can be adopted in non‐Western or resource‐limited settings. Structural and organizational barriers, including high patient‐to‐nurse ratios, time constraints in acute care, and the absence of RCC from most existing nursing leadership competency frameworks, pose significant impediments to the development and expression of RCC in practice and should be explicitly addressed in implementation research. Second, restriction to English‐language publications may have narrowed the cultural breadth of the conceptual findings. Third, the sample of 19 studies is relatively small; while conceptual consistency was achieved with no new attributes emerging from the final studies reviewed, future analyses drawing on a broader literature base may identify additional attributes. Fourth, since concept analysis includes interpretive synthesis, the defining attributes show analytical judgments based on patterns that were seen. Two reviewers agreed on the results, and a systematic audit trail helped reduce interpretive subjectivity, but other ways of thinking about the concepts are still possible. Future empirical studies should validate the defining attributes and conceptual definition of RCC with clinical nursing leaders and managers, testing the framework against practitioner experience and organizational contexts across diverse healthcare systems. A limitation is that RCC shares conceptual territory with therapeutic communication, patient‐centered communication, and relational leadership communication. The boundaries established here are interpretive, and psychometric validation may reveal overlap with adjacent constructs, necessitating refinement. The relative importance of the three attributes may vary across clinical specialties or organizational contexts, and causal relationships between RCC and its identified consequences remain theoretically inferred rather than empirically established. Although older‐adult care was used as an illustrative example, the attributes were identified from 19 studies across varied care settings, supporting the broader relevance of the findings to nursing leadership and management.

Notwithstanding these limitations, this concept analysis provides a theoretically grounded conceptualization of RCC derived through the structured Walker and Avant framework, supporting analytical transparency throughout the analysis process.

## 11. Conclusion

This concept analysis offers an integrated conceptual definition of RCC in nursing leadership and management. The framework brings together relational leadership, communication competence, and person‐centered care as a single relational capability characterized by three interrelated attributes: relational sensitivity, effective information exchange, and collaborative engagement. These attributes tend to co‐occur in practice and, when present together, help characterize RCC in healthcare interactions.

For nursing management, RCC should be recognized as a core relational capability rather than a peripheral skill. It warrants inclusion in competency frameworks, assessment through the behavioral indicators identified, and cultivation through programs that strengthen all three attributes in combination. At the same time, this framework represents a theoretically grounded starting point rather than a fully differentiated construct. Empirical validation across diverse cultural and healthcare organizational contexts is needed to determine whether RCC functions as an independent capability or as a synthesis of existing relational and communicative practices. Future research should test the framework with practicing nurse leaders, develop measurement instruments based on the empirical referents identified, and examine how organizational structures and cultural settings shape RCC in practice.

## 12. Implications for Nursing Management

Nurse managers and leaders require structured programs that build RCC as an integrated capability. Competency frameworks should embed RCC as a unified relational standard, using the behavioral indicators in Table 7 as assessment anchors.

The empirical referents in Table 7 (empathic listening, transparent clinical explanation, verification of shared understanding, and facilitation of collaborative dialog) provide anchors for observation tools and reflective self‐assessment instruments. Simulation‐based learning, interdisciplinary role‐play, and reflective practice offer mechanisms for cultivating all three attributes within the same learning experience. Case‐based learning using the model, borderline, and contrary cases described in this analysis provides a direct pedagogical application. At the organizational level, the antecedents in Table 6 (supportive communication climates, opportunities for structured dialog, and professional development resources) offer a framework for auditing communication policies and identifying structural conditions that enable or inhibit RCC in nursing leadership practice.

## Author Contributions

Joseph Almazan, Darya Zvonareva, Eddieson Pasay‐an, Cris Adolfo, Cyrelle Agunod, and Srinivasa Rao Bolla: study design and conception.

Joseph Almazan, Darya Zvonareva, and Cyrelle Agunod: data acquisition.

Joseph Almazan, Darya Zvonareva, Eddieson Pasay‐an, Cris Adolfo, and Cyrelle Agunod: data analysis and interpretation.

Joseph Almazan, Eddieson Pasay‐an, and Cyrelle Agunod: manuscript writing.

Joseph Almazan, Eddieson Pasay‐an, and Cris Adolfo: supervision.

Joseph Almazan, Darya Zvonareva, Eddieson Pasay‐an, Cris Adolfo, Cyrelle Agunod, and Srinivasa Rao Bolla: critical review and input.

## Funding

This open access publication was supported by Nazarbayev University under the transformative agreement with Wiley.

## Disclosure

All authors reviewed and approved the final version of the manuscript, agreed to be accountable for all aspects of the work, and met at least one of the criteria recommended by the ICMJE (https://www.icmje.org/recommendations/).

## Ethics Statement

This study is a concept analysis based entirely on published literature. It did not involve human participants, animal subjects, or personal data. Formal ethical approval was therefore not required for this study.

## Conflicts of Interest

The authors declare no conflicts of interest.

## Supporting Information

Additional supporting information can be found online in the Supporting Information section.

## Supporting information


**Supporting Information** Supporting Table S1. Database‐specific search strategies, applied filters, and records retrieved for PubMed, APA PsycNet, Scopus, and Web of Science.

## Data Availability

This study is a concept analysis based on published literature. All sources analyzed are cited within the manuscript and are accessible through their respective publishers. No new data were generated or analyzed in this study.
