# Supplementary material for: Relational Communication Competence in Nursing Leadership and Management: A Concept Analysis
Source: J Nurs Manag. 2026 Jul 30;2026:1364784. doi: 10.1155/jonm/1364784 (PMC13422642; doi:10.1155/jonm/1364784)
Supplement: Supplementary file 1 — Supporting Information Supporting Table S1. Database‐specific search strategies, applied filters, and records retrieved for PubMed, APA PsycNet, Scopus, and Web of Science. [file JONM-2026-1364784-s001.zip › Supplementary_Table_S1.June 27.doc]

**Supplementary Table S1.** Database search strategies and PRISMA flow counts

This table provides the database-specific search strategies, limits, and record counts used to support reproducibility of the concept analysis. The flow counts are harmonized with Figure 1.

Search log date: 3 November 2025. Publication window applied for eligibility: January 2000 to December 2024; pre-2000 foundational theoretical works were retained only when directly relevant and agreed by two reviewers.

| Database | Final parenthesized search strategy / platform syntax | Limits / filters applied | Records retrieved |
| --- | --- | --- | --- |
| PubMed | (("relational communication"[All Fields] OR "communication competence"[All Fields]) AND ("nursing leadership"[All Fields] OR "nurse manager"[All Fields] OR "patient-centered care"[All Fields] OR "older adults"[All Fields])) | English language; humans; publication date 2000/01/01 to 2024/12/31; exclude preprints; age filters for aged and aged 80 and over where available; eligible publication types included observational, evaluation, interview, comparative, review, scoping review, systematic review, and meta-analysis records. | 3,024 |
| APA PsycNet | ((Any Field: "communication competence" OR Any Field: healthcare) AND (Any Field: patient)) | Peer-reviewed journals only; publication years 2000-2024; English-language records where available. | 54 |
| Scopus | TITLE-ABS-KEY(("relational communication" OR "communication competence") AND ("nursing leadership" OR "nurse manager" OR "patient-centered care" OR "older adults")) | Document type: article; source type: journal; language: English; publication stage: final; publication years 2000-2024. | 1,212 |
| Web of Science | ((ALL=("relational communication") OR ALL=("communication competence")) AND (ALL=("nursing leadership") OR ALL=("nurse manager") OR ALL=("patient-centered care") OR ALL=("older adults"))) | Document type: article; publication years 2000-2024; English-language records where available. | 27 |

**PRISMA flow counts**

| PRISMA item | n | Notes |
| --- | --- | --- |
| Records identified from databases | 4,317 | PubMed n = 3,024; Scopus n = 1,212; Web of Science n = 27; APA PsycNet n = 54. |
| Duplicate records removed before screening | 12 | Duplicates removed in Zotero before title/abstract screening. |
| Records screened | 4,305 | After removal of duplicates. |
| Records excluded at title/abstract screening | 4,223 | Records did not meet conceptual or contextual screening criteria. |
| Reports sought for retrieval | 82 | Full-text reports sought for eligibility assessment. |
| Reports not retrieved | 7 | Full text unavailable during retrieval. |
| Reports assessed for eligibility | 75 | Full-text reports assessed against inclusion/exclusion criteria. |
| Reports excluded after full-text assessment | 56 | Technical/information-transfer focus only (n = 21); outside healthcare context (n = 12); insufficient conceptual detail/no communication dynamics (n = 23). |
| Reports of included studies | 19 | Studies included in the concept analysis. |

**Reviewer-facing note:** The search strategies above are fully parenthesized for interpretability. Counts reflect the search log and may differ if searches are rerun after database indexing updates.
